# Draft genome sequence of Actinopolyspora saharensis DSM 46666, a rare actinomycete isolated from the Algerian Sahara

**DOI:** 10.1099/acmi.0.001099.v3

**Published:** 2026-02-13

**Authors:** Shuangqing Zhou, Rafika Saker, Noureddine Bouras, Guendouz Dif, Yvonne Mast, Imen Nouioui

**Affiliations:** 1 Leibniz-Institute DSMZ – German Collection of Microorganisms and Cell Cultures, Inhoffenstraße 7B, 38124 Braunschweig, Germany; 2 College of Pharmacy, Guilin Medical University, Guilin 541199, PR China; 3 Laboratoire de Biologie des Systèmes Microbiens (LBSM), Ecole Normale Supérieure Cheikh Mohamed El Bachir El Ibrahimi, BP 92, Algiers 16050, Algeria; 4 Département des Sciences de la Nature et de la Vie, Faculté des Sciences, Université d’Alger 1, Algiers 16000, Algeria; 5 Laboratoire de Valorisation et Conservation des Ecosystèmes Arides (LVCEA), Faculté des Sciences de la Nature et de la Vie et Sciences de la Terre, Université de Ghardaia, BP 455, Ghardaïa 47000, Algeria; 6 Département des Sciences Naturelles, École Normale Supérieure Taleb Abderrahmane de Laghouat, BP 4033, Laghouat 03000, Algeria; 7 Braunschweig Integrated Centre of Systems Biology (BRICS), Rebenring 563, 8106 Braunschweig, Germany; 8 Technische Universität Braunschweig, Institut für Mikrobiologie, Rebenring 563, 8106 Braunschweig, Germany

**Keywords:** antibiotic, extreme habitat, genome mining, rare actinomycetes, whole-genome sequence

## Abstract

*Actinopolyspora saharensis* DSM 46666 (=H244), a rare actinomycete, was isolated from Palm grove in the Oasis of Inghid in the Mzab region of the Algerian Sahara and deposited in the German Collection of Microorganisms and Cell Cultures (DSMZ). Here, we report the draft genome sequence of strain DSM 46666, with a size of 4.67 Mbp and a G+C content of 69.5 %. Bioinformatic analysis of the genomic sequence revealed the carbohydrate-active enzyme gene repertoires involved in carbohydrate metabolism and the occurrence of 14 biosynthetic gene clusters disclosing the secondary metabolite capacity of strain DSM 46666.

## Data Summary

This Whole Genome Shotgun project has been deposited at DDBJ/ENA/GenBank under the accession number JBPQHB000000000. The version described in this paper is version JBPQHB000000000. The raw sequence reads have been submitted to Sequence Read Archive at NCBI (SRX31383543). The 16S rRNA gene sequence of *Actinopolyspora saharensis* DSM 46666 has been deposited at GenBank under the accession number KJ574187.

## Announcement

The genus *Actinopolyspora* of the family *Pseudonocardiaceae* comprises 12 validly named species, with *Actinopolyspora halophila* as the type species (https://lpsn.dsmz.de/genus/actinopolyspora) [1]. Members of this genus are considered rare actinomycetes and have been isolated from saline, hypersaline and alkaline environments and are known to produce bioactive compounds with antimicrobial, antitumour and biofortification properties [2,4]. However, little is known about their ecological diversity and functional capabilities. Comparative genomic analysis reveals that the enzymatic activity associated with dienelactone hydrolase is highly conserved within the species of *Actinopolyspora saharensis*, despite the different geographical and ecological origins of the strains [5]. In view of investigating the German Culture Collection (DSMZ) for a rare halophilic actinobacterial strain, the genome of *A. saharensis* DSM 46666 was sequenced and its biosynthetic potential was analysed based on genome mining appraoch.

*A. saharensis* DSM 46666 (=H244) was isolated from a desert soil sample collected from Palm grove in the Oasis of Inghid, Béni-Isguen, Ghardaia, Mzab region, Algeria, as described by Meklat *et al.* [6] and deposited at the DSMZ culture collection. The strain was cultivated in DSMZ 1504 medium supplemented with 15% of NaCl and incubated at 28 °C for 14 days. Under this growth condition, it had white aerial mycelium and beige substrate mycelium, as shown in the DSMZ online catalogue (https://www.dsmz.de/collection/catalogue/details/culture/DSM-46666). DNA extraction and whole-genome sequencing of the strain were carried out by MicrobesNG service [Birmingham, UK, (https://microbesng.com/)]. In total, 50 mg of wet biomass was suspended in 500 µl of DNA/RNA Shield buffer (Zymo Research, California, USA) and sent to the sequencing service. The bacterial suspension was lysed with 120 µl of Tris/EDTA buffer supplemented with proteinase K (VWR Chemicals, Ohio, USA) and SDS (Sigma-Aldrich, Missouri, USA) at final concentrations of 0.1 mg ml^−1^ and 0.5% (v/v), respectively. The lysate was incubated for 5 min at 65 °C. An equal volume of solid-phase reversible immobilization beads was used for the purification of genomic DNA, which was resuspended in EB buffer (10 mM Tris/HCl, pH 8.0). DNA quantification was measured using the Quant-iT dsDNA HS assay (Thermo Fisher Scientific) on an Eppendorf AF2200 plate reader (Eppendorf UK Ltd., UK). Genomic libraries were prepared with the Nextera XT Library Prep Kit (Illumina, San Diego, California, USA) according to the manufacturer’s instructions with the following modifications: the DNA was increased twofold and the PCR elongation time was increased to 45 s. A Hamilton Microlab STAR automated liquid handling system (Hamilton Bonaduz AG, Switzerland) was used for library preparation. Genome sequencing was carried out on an Illumina NovaSeq 6000 platform (Illumina, San Diego, USA) with 2×250 bp paired-end reads, achieving ~53× coverage. Read trimming was carried out with Trimmomatic (v0.3) [7] with a window quality cutoff of Q15. *De novo* assembly was carried out using SPAdes v3.7 [8]. Prokka v1.11 was used for contig annotation [9]. The authenticity of the strain was confirmed by comparing the 16S rRNA gene sequence obtained by PCR with the corresponding sequence extracted from the assembled genome. The genome sequence was submitted to GenBank under accession number JBPQHB000000000 and automatically annotated via NCBI Prokaryotic Genome Annotation Pipeline (PGAP) [10,12].

**Fig. 1.**
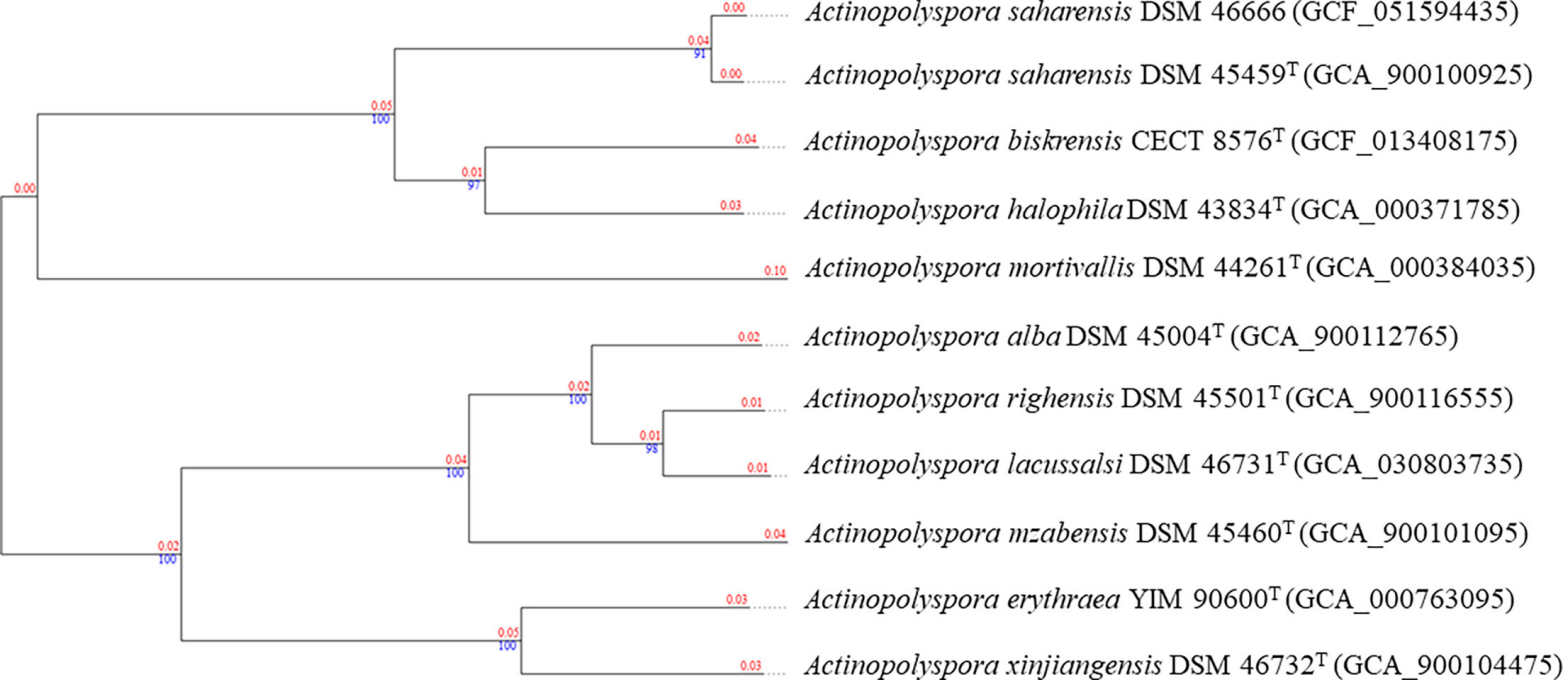
Genome Blast Distance Phylogeny (GBDP) tree inferred with FastME 2.1.6.1 from GBDP distances calculated from genome sequences, illustrating the evolutionary relationship between *A. saharensis* DSM 46666 and *Actinopolyspora* species validly named. The branch lengths are scaled according to the GBDP distance formula d5. The blue numbers are GBDP pseudo-bootstrap support values >60% from 100 replications, with an average branch support of 98.0%. The red numbers are the branch length values.

The final assembly of strain DSM 46666 revealed a genome size of 4.7 Mbp, with a G+C content of 69.5 mol%, 4,058 CDSs, 73 RNAs, an N50 of 247.4 kb and an L50 of 7 (Table 1). Taxonomic assignment placed the strain within the genus *Actinopolyspora*, based on 16S rRNA gene sequence, which showed 99.8% identity to *A. saharensis* DSM 45459^T^, as determined using the EzBioCloud server [13]. The phylogenetic relatedness of DSM 46666 to validly named *Actinopolyspora* species was further assessed through taxogenomic analyses using the type strain genome server (TYGS) [14]. Strain DSM 46666 formed a well-supported subclade with *A. saharensis* DSM 45459^T^, adjacent to a subclade containing *Actinopolyspora biskrensis* and *A. halophila* (Fig. 1). Digital DNA–DNA hybridization (dDDH) and average nucleotide identity (ANI) values between strain DSM 46666 and its close phylogenomic neighbour, *A. saharensis* DSM 45459^T^, were determined using the TYGS and the EzBioCloud servers [15], respectively. The average aligned length for ANI between the studied genomes was 3,194,858 pb. The dDDH (95.5%) and ANI (99.1%) values were above the established species delineation thresholds of 70% (dDDH) and 95–96% (ANI), confirming that strain DSM 46666 belongs to the *A. saharensis* species [16,19].

**Table 1.** Genomic features of *A. saharensis* DSM 46666 and the type strain *A. saharensis* DSM 45459^T^

	Strain DSM 46666	Strain DSM 45459**^T^**
Genome size (Mb)	4.7	4.7
G+C content (mol%)	69.5	69.5
CDSs	4,058	4,052
RNA numbers	73	72
N50	247.4 kb	2.6 Mb
L50	7	1
Contigs	70	2
Coverage	53×	356×
BioSample	SAMN49880177	SAMN04489718
BioProject	PRJNA1289121	PRJEB15893
Completeness (%)	97.98	96.91
Contamination (%)	1.88	1.65
Accession number	JBPQHB000000000	NZ_FNKO00000000.1

**Fig. 2.**
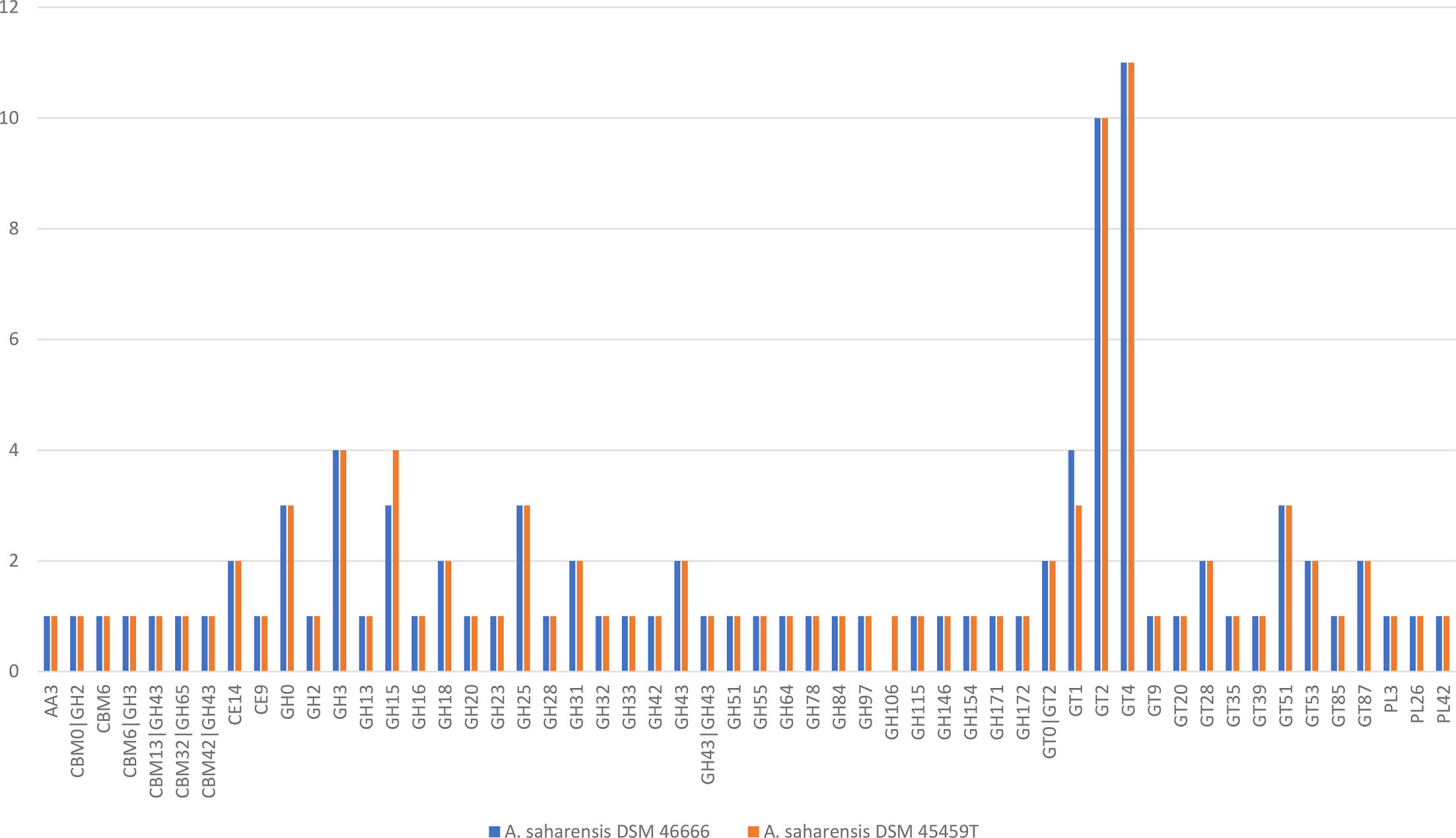
Comparison of predicted CAZymes of two strains of *A. saharensis* (DSM 46666 and DSM 45459^T^) using dbCAN3 server. Only CAZymes predicted by HMMER, DIAMOND and Hotpep tools were considered. AA: auxiliary activities; CBM: carbohydrate-binding modules; CE: carbohydrate esterases; GH: glycoside hydrolases; PL: polysaccharide lyases; GT: glycosyl transferases.

The abundance and distribution of carbohydrate-active enzyme (CAZyme) families and their domains in the genome sequence of strain DSM 46666 and DSM 45459^T^ were assessed across the analysed genomes using the dbCAN3 platform (https://bcb.unl.edu/dbCAN2/) [20,21]. The outcome of this analysis provides a better understanding of the role of these strains in ecosystems and can be used to further study research aimed at exploiting the biotechnological potential of these strains through enzyme engineering. To ensure reliability, a CAZyme family was considered present only if it was independently confirmed by all three integrated tools, HMMER [22,23], DIAMOND [24,25] and Hotpep [26], which work in a complementary manner. The annotation with dbCAN was performed using three algorithms with very conservative thresholds: DIAMOND (e-value<1×10⁻¹⁰²), HMMER (e-value<1×10⁻¹⁷ and coverage>0.45) and Hotpep (frequency>2.6 and number of hits>6). HMMER utilizes hidden Markov models to identify conserved sequence profiles, DIAMOND performs fast similarity searches against reference CAZyme databases and Hotpep detects conserved short peptide motifs characteristic of CAZymes. To enhance the reliability of the annotations, only genes confirmed by all three tools were retained, following a consensus-based scoring approach. The CAZyme repertoires of the two *A. saharensis* strains are highly similar, with a total of 94 CAZyme genes in strain DSM 46666 and 95 in strain DSM 45459^T^ (Fig. 2). This represents ~2% of the total CDSs in each genome, highlighting a consistent investment in carbohydrate metabolism across both strains. The accession numbers for the predicted CAZyme protein sequences of strain DSM 46666 were listed in Table S1 (available in the online Supplementary Material). These findings expand our knowledge of the biological roles and potential applications of CAZymes of this taxon.

Rare halophilic actinomycetes, such as *Actinopolyspora* strains, are known for their significant hydrolytic activities and metabolic adaptations to harsh saline environments [27,28], which could reflect their bioremediation potential. Bouras *et al.* [5] studied the ecological diversity and functional capacities of *A. saharensis* strains based on comparative genomic analyses, focusing mainly on the genetic ability of the type strain H32^T^ (Algeria) and strain BKK2 (China) to produce enzymes (dienelactone hydrolase) capable of degrading petroleum-based plastics. This study showed that the carbohydrate enzymatic activities of *A. saharensis* strains are highly conserved, despite their ecological diversity. *A. saharensis* strains have genetic ability to produce potential valuable biocatalyst that may be useful in tackling ecological plastic pollution [4].

## Supplementary material

10.1099/acmi.0.001099.v3 Supplementary Material 1.

## Abbreviations

ANI: average nucleotide identity
CAZyme: carbohydrate-active enzyme
dDDH: digital DNA–DNA hybridization
DSMZ: Deutsche Sammlung von Mikroorganismen und Zellkulturen
GBDP: genome-based distance phylogeny
TYGS: type strain genome server
