# Supplementary material for: Draft genome sequence of Actinopolyspora saharensis DSM 46666, a rare actinomycete isolated from the Algerian Sahara
Source: Access Microbiol. 2026 Feb 13;8(2):001099.v3. doi: 10.1099/acmi.0.001099.v3 (PMC12904601; doi:10.1099/acmi.0.001099.v3)
Supplement: Supplementary Material 1. [file acmi-8-01099-s001.pdf]

Draft Genome Sequence of *Actinopolyspora saharensis* DSM 46666, a Rare Actinomycete Isolated from the Algerian Sahara

Shuangqing Zhou[1,2], Rafika Saker[3,4], Noureddine Bouras[3,5], Guendouz Dif[3,6], Yvonne Mast[1,7,8], Imen Nouioui[1]*

Affiliations

[1]Leibniz-Institute DSMZ – German Collection of Microorganisms and Cell Cultures, Inhoffenstraße 7B, 38124 Braunschweig, Germany
[2]College of Pharmacy, Guilin Medical University, Guilin, 541199, PR China
[3]Laboratoire de Biologie des Systèmes Microbiens (LBSM), Ecole Normale Supérieure Cheikh Mohamed El Bachir El Ibrahimi, BP 92, Kouba, Algiers, Algeria
[4]Département des Sciences de la Nature et de la Vie, Faculté des Sciences, Université d'Alger 1, Algeria
[5]Laboratoire de Valorisation et Conservation des Ecosystèmes Arides (LVCEA), Faculté des Sciences de la Nature et de la Vie et Sciences de la Terre, Université de Ghardaia, B.P. 455, Ghardaïa, Algeria
[6]École Normale Supérieure Taleb Abderrahmane de Laghouat, Département des Sciences Naturelles, BP 4033, Laghouat 03000, Algeria
[7]Braunschweig Integrated Centre of Systems Biology (BRICS), Rebenring 56, 38106 Braunschweig, Germany
[8]Technische Universität Braunschweig, Institut für Mikrobiologie, Rebenring 56, 38106 Braunschweig, Germany

Corresponding author: Imen Nouioui, imen.nouioui@dsmz.de

Table S1. Predicted carbohydrate-active enzymes of *Actinopolyspora saharensis* DSM 46666 using the CAZy Database and dbCAN3 Tools (HMMER, DIAMOND, and Hotpep). Only CAZymes predicted by all three tools were considered

Protein abbreviation	Protein family	Protein length(aa)	Protein accession number
GH43_34	Glycoside Hydrolase Family 43 / Subf 34	420	WP_092520966.1
GH11	Glycoside Hydrolase Family 11	230	WP_092521071.1
AA3_4	Auxiliary Activity Family 3 / Subf 4	556	WP_092521163.1
GT83	GlycosylTransferase Family 83	766	WP_092521279.1
GT2	GlycosylTransferase Family 2	302	WP_092521427.1
GT9	GlycosylTransferase Family 9	338	WP_092521439.1
GT4	GlycosylTransferase Family 4	406	WP_092521441.1
GH43_5	Glycoside Hydrolase Family 43 / Subf 5	326	WP_092521471.1
GT1	GlycosylTransferase Family 1	394	WP_092521652.1
AA6	Auxiliary Activity Family 6	204	WP_092521750.1
GT4	GlycosylTransferase Family 4	404	WP_092521914.1
CE4	Carbohydrate Esterase Family 4	257	WP_092521923.1
GH178	Glycoside Hydrolase Family 178	366	WP_092521937.1
GH25	Glycoside Hydrolase Family 25	481	WP_092521943.1
GT1	GlycosylTransferase Family 1	402	WP_092522116.1
GH13	Glycoside Hydrolase Family 13	434	WP_092523406.1
GH13	Glycoside Hydrolase Family 13	417	WP_092523408.1
GT2	GlycosylTransferase Family 2	292	WP_092523956.1
GT4	GlycosylTransferase Family 4	384	WP_092523958.1
GH179	Glycoside Hydrolase Family 179	388	WP_092524119.1
GT4	GlycosylTransferase Family 4	397	WP_092524290.1
GH15	Glycoside Hydrolase Family 15	853	WP_092524530.1
AA10	Auxiliary Activity Family 10	202	WP_092524980.1
AA7	Auxiliary Activity Family 7	442	WP_092525024.1
GH15	Glycoside Hydrolase Family 15	625	WP_092525512.1
CE14	Carbohydrate Esterase Family 14	293	WP_092526596.1
GH13_32	Glycoside Hydrolase Family 13 / Subf 32	494	WP_142077745.1
GH25	Glycoside Hydrolase Family 25	259	WP_165631551.1
GH3	Glycoside Hydrolase Family 3	760	WP_165631588.1
GT4	GlycosylTransferase Family 4	431	WP_165631924.1
AA3_2	Auxiliary Activity Family 3 / Subf 2	522	WP_165632225.1
GH3	Glycoside Hydrolase Family 3	909	WP_165632255.1
GT119	GlycosylTransferase Family 119	480	WP_165632331.1
GH188	Glycoside Hydrolase Family 188	362	WP_165632654.1

GT20	GlycosylTransferase Family 20	479	WP_165632809.1
GT83	GlycosylTransferase Family 83	504	WP_165632864.1
GT35	GlycosylTransferase Family 35	850	WP_165633440.1
AA7	Auxiliary Activity Family 7	500	WP_165633510.1
GT2	GlycosylTransferase Family 2	840	WP_165633646.1
GT51	GlycosylTransferase Family 51	792	WP_165633716.1
AA7	Auxiliary Activity Family 7	446	WP_165633765.1
GH42	Glycoside Hydrolase Family 42	683	WP_165633873.1
GT28	GlycosylTransferase Family 28	380	WP_175455021.1
GH25	Glycoside Hydrolase Family 25	273	WP_175455124.1
GH28	Glycoside Hydrolase Family 28	475	WP_207630390.1
GT2	GlycosylTransferase Family 2	282	WP_207630733.1
GT9	GlycosylTransferase Family 9	319	WP_217637721.1
GH183	Glycoside Hydrolase Family 183	326	WP_245695602.1
GT51	GlycosylTransferase Family 51	690	WP_245695767.1
GT4	GlycosylTransferase Family 4	411	WP_245695797.1
GT87	GlycosylTransferase Family 87	420	WP_245695837.1
PL3_4	Polysaccharide Lyase Family 3 / Subf 4	268	WP_281241052.1
GT51	GlycosylTransferase Family 51	1201	WP_281241075.1
PL3_4	Polysaccharide Lyase Family 3 / Subf 4	263	WP_342751246.1
GT2	GlycosylTransferase Family 2	704	WP_347567553.1
CE14	Carbohydrate Esterase Family 14	304	WP_438386970.1
GH172	Glycoside Hydrolase Family 172	880	WP_438386994.1
GH23	Glycoside Hydrolase Family 23	397	WP_438387016.1
GH23	Glycoside Hydrolase Family 23	277	WP_438387019.1
GH23	Glycoside Hydrolase Family 23	265	WP_438387020.1
AA3	Auxiliary Activity Family 3	571	WP_438387066.1
GH2_10	Glycoside Hydrolase Family 2 / Subf 2	1275	WP_438387072.1
GH20	Glycoside Hydrolase Family 20	548	WP_438387287.1
GT2	GlycosylTransferase Family 2	469	WP_438387303.1
GH18	Glycoside Hydrolase Family 18	381	WP_438387304.1
GT4	GlycosylTransferase Family 4	390	WP_438387306.1
GT4	GlycosylTransferase Family 4	412	WP_438387316.1
GH18	Glycoside Hydrolase Family 18	342	WP_438387334.1
GT85	GlycosylTransferase Family 85	665	WP_438387427.1
GH115	Glycoside Hydrolase Family 115	1044	WP_438387646.1
GH43_24+CBM13	Glycoside Hydrolase Family 43 / Subf 24+Carbohydrate-Binding Module Family 13	490	WP_438387665.1
GH5	Glycoside Hydrolase Family 5	396	WP_438387666.1
PL42	Polysaccharide Lyase Family 42	478	WP_438387668.1
GH154	Glycoside Hydrolase Family 154	657	WP_438387669.1

AA3	Auxiliary Activity Family 3	519	WP_438387717.1
GH31_2	Glycoside Hydrolase Family 31 / Subf 2	839	WP_438387721.1
GH106	Glycoside Hydrolase Family 106	986	WP_438387725.1
GH78	Glycoside Hydrolase Family 78	1069	WP_438387726.1
GH55_1	Glycoside Hydrolase Family 55 / Subf 1	600	WP_438387733.1
GH84	Glycoside Hydrolase Family 84	686	WP_438387886.1
GH33	Glycoside Hydrolase Family 33	378	WP_438387962.1
GT2	GlycosylTransferase Family 2	417	WP_438387972.1
GT2	GlycosylTransferase Family 2	393	WP_438387976.1
GH64	Glycoside Hydrolase Family 64	392	WP_438387984.1
PL26	Polysaccharide Lyase Family 3 / Subf 26	914	WP_438388003.1
GH16_3	Glycoside Hydrolase Family 16 / Subf 3	301	WP_438388009.1
GH65	Glycoside Hydrolase Family 65	901	WP_438388046.1
GT53	GlycosylTransferase Family 53	1034	WP_438388053.1
GT53	GlycosylTransferase Family 53	1122	WP_438388054.1
GH25	Glycoside Hydrolase Family 25	339	WP_438388083.1
GH171	Glycoside Hydrolase Family 171	428	WP_438388085.1
GH3	Glycoside Hydrolase Family 3	595	WP_438388086.1
GT76	GlycosylTransferase Family 76	373	WP_438388119.1
GH184	Glycoside Hydrolase Family 184	327	WP_438388199.1
GT83	GlycosylTransferase Family 83	486	WP_438388219.1
GT28	GlycosylTransferase Family 28	831	WP_438388274.1
GT4	GlycosylTransferase Family 4	378	WP_438388339.1
GT39	GlycosylTransferase Family 39	525	WP_438388427.1
GT2	GlycosylTransferase Family 2	393	WP_438388429.1
GH51_2	Glycoside Hydrolase Family 51 / Subf 2	680	WP_438388452.1
GH43_26+CBM42	Glycoside Hydrolase Family 43 / Subf 26+Carbohydrate-Binding Module Family 42	495	WP_438388453.1
GT4	GlycosylTransferase Family 4	373	WP_438388460.1
GT2	GlycosylTransferase Family 2	309	WP_438388464.1
GT4	GlycosylTransferase Family 4	365	WP_438388466.1
CBM6	Carbohydrate-Binding Module Family 6	1424	WP_438388487.1
GT119	GlycosylTransferase Family 119	484	WP_438388525.1
GT2	GlycosylTransferase Family 2	1071	WP_438388538.1
GH3	Glycoside Hydrolase Family 3	618	WP_438388545.1
GH15	Glycoside Hydrolase Family 15	716	WP_438388563.1
CE4	Carbohydrate Esterase Family 4	236	WP_438388635.1
CE14	Carbohydrate Esterase Family 14	245	WP_438388638.1

GH3+CBM6	Glycoside Hydrolase Family 3+Carbohydrate-Binding Module Family 6	1009	WP_438388656.1
GH31_4	Glycoside Hydrolase Family	667	WP_438388657.1
GH12	Glycoside Hydrolase Family 12	230	WP_438388658.1
GH32	Glycoside Hydrolase Family 32	518	WP_438388690.1
GT2	GlycosylTransferase Family 2	263	WP_438388695.1
GH97	Glycoside Hydrolase Family 97	714	WP_438388741.1
CE9	Carbohydrate Esterase Family 9	384	WP_438388752.1
GT1	GlycosylTransferase Family 1	386	WP_438388779.1
GH146	Glycoside Hydrolase Family 146	933	WP_438388826.1
GH43_22	Glycoside Hydrolase Family 43 / Subf 22	478	WP_438388828.1
GH2_18	Glycoside Hydrolase Family 2 / Subf 18	618	WP_438388852.1
GT87	GlycosylTransferase Family 87	423	WP_438388920.1
CE4	Carbohydrate Esterase Family 4	220	WP_438388922.1
GT87	GlycosylTransferase Family 87	394	WP_438388939.1
CAZYme gene	94		
% CAZome	2.05		
